# Whole transcriptome sequence data of 5-FU sensitive and 5-FU resistant tumors generated in a mouse model of de novo carcinogenesis

**DOI:** 10.1016/j.dib.2018.08.209

**Published:** 2018-09-07

**Authors:** Demetris Iacovides, Charalambos Loizides, Georgios Mitsis, Katerina Strati

**Affiliations:** a Department of Biological Sciences, University of Cyprus, Cyprus; b Center for Intelligent Systems and Networks, University of Cyprus, Cyprus

## Abstract

We have performed whole transcriptome sequencing of 5-FU resistant and 5-FU sensitive tumors generated in a mouse model of de novo carcinogenesis that closely recapitulates tumor initiation, progression and maintenance in vivo. Tumors were generated using the DMBA/TPA model of chemically induced carcinogenesis [1], tumor-bearing mice were subsequently treated with 5-FU, and tumor growth as well as response to treatment was monitored by measuring tumor volume twice a week. Based on these measurements, we selected two 5-FU resistant and two 5-FU sensitive tumors and performed whole transcriptome sequencing and in order to identify differentially expressed transcripts between the two sets. Data obtained is deposited and available through NCBI SRA (reference number SRP155180 – https://www.ncbi.nlm.nih.gov/sra/?term=SRP155180).

**Specifications table**

**Table:** 

Subject area	*Biology*
More specific subject area	*Mus Musculus - Skin Tumors*
Type of data	*Table, figures, raw sequence reads*
How data was acquired	*Illumina HiSeq™ 2000*
Data format	Raw and analyzed
Experimental factors	*5-FU sensitive VS 5-FU resistant tumors*
Experimental features	*Mouse model of chemically-induced carcinogenesis. Tumors were induced with DMBA/TPA and mice were treated intraperitoneally with 5-fluorouracil. 5-FU-sensitive and resistant tumors were harvested and processed for RNA extraction. Whole transcriptome sequencing was performed on sensitive and resistant tumors using* Illumina HiSeq™ 2000.
Data source location	*Nicosia, Cyprus*
Data accessibility	*Data is with this article and available at*
	https://www.ncbi.nlm.nih.gov/sra/?term=SRP155180
Related research article	[5] Loizides C, Iacovides D, Hadjiandreou M. M, Rizki G, Achilleos A, Strati K, and Mitsis G. D. Model-based tumor growth dynamics and therapy response in a mouse model of de novo carcinogenesis, PloS One, 10(12), 2015, p. e0143840.
	https://doi.org/10.1371/journal.pone.0143840

**Value of the data**

• Data in this article can be used to identify differentially expressed genes between tumors that are sensitive or resistant to treatment with 5-FU.
• These transcripts represent potential markers of response to treatment with 5-FU. Thus, data presented herein could facilitate the development of clinically meaningful biomarker signatures of response to 5-FU and contribute towards personalized cancer treatment.

## 1 Data

**Fig. 1:**
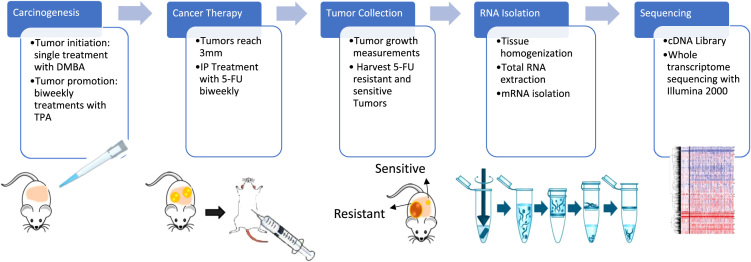
Generation of 5-FU sensitive and resistant tumors in a mouse model of chemically-induced carcinogenesis. Carcinogenesis was initiated with DMBA, followed by bi-weekly treatments with TPA to promote tumor growth. Isolated RNA from 2 pairs of 5-FU sensitive and 5-FU resistant tumors was used to perform whole-transcriptome sequencing using Illumina^2000^, in order to identify differentially expressed genes between sensitive and resistant tumors.

**Fig. 2:**
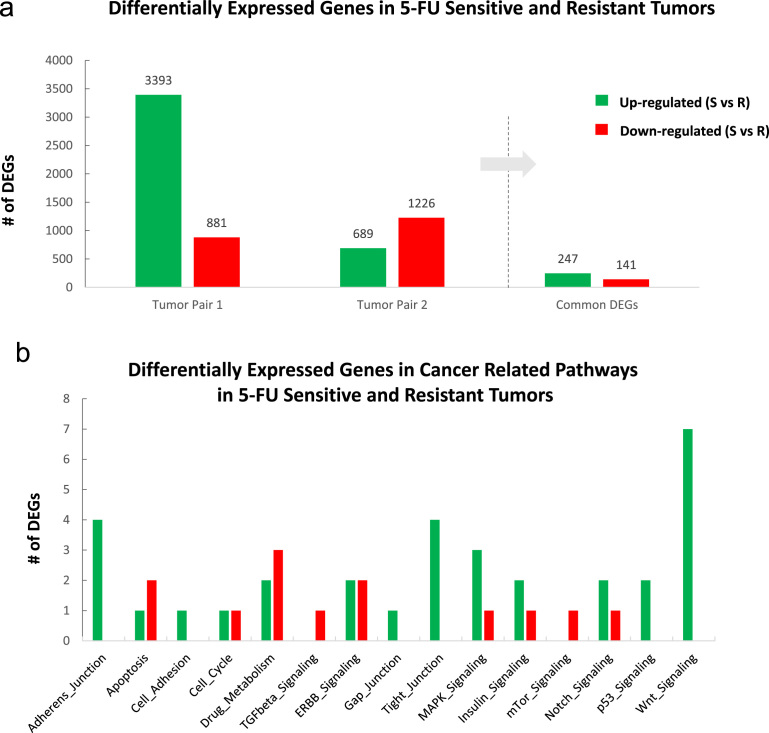
(A) Total number of differentially expressed genes within each tumor pair. Comparative analysis was performed as sensitive (S) vs resistant (R) tumor. In total, 247 transcripts are commonly upregulated in sensitive tumors compared to resistant tumors, and 141 transcripts are commonly downregulated in sensitive tumors compared to resistant tumors (B) S vs R DEGs in cancer-related molecular pathways.

Eight raw sequence read data files are shared. Two 5-FU sensitive (S1 and S2) and two 5-FU resistant (NR1 and NR2) tumors were sequenced, and each tumor is represented with two technical replicates (A and B) (Fig. 1, Fig. 2).

## 2 Experimental design, materials and methods

### 2.1 Breeding and genotyping

In order to accelerate tumor formation, we used transgenic mice expressing the HPV E6/E7 viral oncogenes under the keratin-14 promoter [2], [3]. These mice were obtained by crossing K14E6H females with K14E7h heterozygous females (K14E6hK14E7h). To confirm presence of the E6 and E7 transgenes, we extracted DNA for mouse tail and performed PCR genotyping, using Sigma DNA extraction Kit (Sigma-Aldrich, cat. # G1N10) and KAPATaq (Kappa Biosystems, cat. # KK1015). (K14709-4/E7TTL) and (Oligo2/E6TTL) primers were used to detect E6 and E7 respectively, as previously described [4].

### 2.2 DMBA/TPA treatment

Treatment with 200 μl of 0.03 μmol/μl DMBA (Sigma-Aldrich, cat. # D3254) was administered once on the back of 7–8 weeks old mice previously shaved at the area. Two weeks after DMBA administration, we initiated treatment with 2.5 μg TPA diluted in 200 μl acetone twice a week (Sigma-Aldrich, cat. #P8139) until a mouse was sacrificed.

### 2.3 Treatment with 5-Fluorouracil

Treatment with 50 mg/kg of 5-FU was initiated when the first tumor on a mouse reached 3–4 mm in size. The drug was administered intraperitonially (5-FU; Sigma-Aldrich, cat. # F6627) once a week, until any tumor on the animal reached ~1 cm in diameter, at which point the mouse was sacrificed.

### 2.4 Tumor measurements

Measurements of tumor volume on a mouse were recorded twice a week during treatment with 5-FU, as previously described [5]. As tumors generated here had an ellipsoid shape, tumor volume (*V*) was calculated using the formula:

$$V=\frac{\pi }{6}{\left(\mathrm{xy}\right)}^{\frac{3}{2}}$$

where *x* and *y* denote the length and width of the tumor respectively [6].

In order to determine sensitivity and resistance to 5-FU treatment, we compared tumor volume measurements obtained right before commencing treatment with 5-FU with volumes of the same tumors just right before sacrificing the animal. Sensitive tumors were selected based on at least 50% reduction in tumor size, and resistant tumors were selected as those with at least 2-fold growth in volume despite treatment with 5-FU. One sensitive and one resistant tumor were selected from each animal for sequencing.

### 2.5 Transcriptomic profiling

Total RNA was extracted from 5-FU sensitive and resistant mouse tumors using the RNeasy kit (Qiagen, cat. # 74104), DNase-treated with TURBO DNA-free kit (Ambion,cat. # AM1907) and quantified using NanoDrop. RNA purity was assessed by A260/A280 and A260/230 ratios (>1.8) using Nanodrop.

Magnetic beads with Oligo (dT) were subsequently used to isolate mRNA which was then fragmented into short fragments, purified and resolved for end reparation and single nucleotide A (adenine) addition. The short mRNA fragments were connected with adapters, subjected to agarose gel electrophoresis, and cDNA was subsequently synthesized using the mRNA fragments as templates through PCR amplification. Agilent 2100 Bioanaylzer and ABI StepOnePlus Real-Time PCR System were used for quantification and qualification of the sample library, and the library was then sequenced using Illumina HiSeq™ 2000. Sequence alignment and quantification analysis of gene expression was done using SOAPaligner/SOAP2 [7]. Transcript reads were assembled using Cufflink [8]. KEGG [9] was used to perform pathway enrichment analysis of differentially expressed genes (DEGs), and expression pattern analysis was performed using Cluster [10] and JavaTreeView [11]. Gene expression levels were measured and normalized using the RPKM method [12]. For each pair of sensitive and resistant tumor samples, we then calculated the log2-ratio of each gene׳s corresponding RPKM value, and subsequently calculated the p-value for the test that the expression levels between the two samples for a specific gene are different. Since multiple comparisons were performed, we corrected these *p*-values using the Benjamini and Yekutieli method [13]. We consider significant DEGs those with adjusted *p* values less than 1e^−4^.
